# The Role of TGFβ and Other Cytokines in Regulating Mast Cell Functions in Allergic Inflammation

**DOI:** 10.3390/ijms231810864

**Published:** 2022-09-17

**Authors:** Tamara T. Haque, Pamela A. Frischmeyer-Guerrerio

**Affiliations:** Laboratory of Allergic Diseases, National Institute of Allergy and Infectious Diseases, National Institutes of Health, Bethesda, MD 20892, USA

**Keywords:** mast cells, allergy, TGFβ, cytokines, inflammation

## Abstract

Mast cells (MC) are a key effector cell in multiple types of immune responses, including atopic conditions. Allergic diseases have been steadily rising across the globe, creating a growing public health problem. IgE-mediated activation of MCs leads to the release of potent mediators that can have dire clinical consequences. Current therapeutic options to inhibit MC activation and degranulation are limited; thus, a better understanding of the mechanisms that regulate MC effector functions in allergic inflammation are necessary in order to develop effective treatment options with minimal side effects. Several cytokines have been identified that play multifaceted roles in regulating MC activation, including TGFβ, IL-10, and IL-33, and others that appear to serve primarily anti-inflammatory functions, including IL-35 and IL-37. Here, we review the literature examining cytokines that regulate MC-mediated allergic immune responses.

## 1. Introduction

Mast cells are innate, sentinel cells that reside in tissue sites at the interface of the host’s external environment. They were first discovered by Paul Ehrlich in 1878. Evolutionarily, these cells provide protection against parasitic, bacterial and viral infections, tumor growth, and snake venom poisoning [1,2,3,4,5]. Due to the rapid rise in allergic disorders in industrialized nations, mast cells have most extensively been studied in the context of allergic diseases, given their central role as effector cells in type-2 inflammatory responses [6]. Mast cells express the high-affinity IgE receptor, FcεRI. Upon ligation of IgE bound to FcεRI by allergen, intracellular signaling is initiated with phosphorylation of the immunoreceptor tyrosine-based activation motifs (ITAMs) in the intracellular domain of FcεRI by Lyn [7]. Lyn and Fyn are further recruited and phosphorylated, followed by Syk recruitment to the ITAMs region, which serves as a Syk docking site [8]. Syk then activates LAT, which serves as an adaptor for several signaling proteins, leading to the activation of Bkt and PLCγ [9]. These events culminate in the release of preformed mediators contained in granules, such as histamine and proteases, within minutes, as well as lipid mediators and newly synthesized mediators including cytokines, within hours, thus displaying a multi-phasic response corresponding to specific signaling events downstream of FcεRI [8,9]. FcεR1 signaling also induces multiple pathways that downregulate its activities, the most well-studied being the autoregulatory induction of phosphatases such as SH2-containing inositol phosphatase (SHIP) and SH2-containing phosphatase1 (SHP-1). Mast cells also highly express the IL-33 receptor ST2 and other cytokine receptors, allowing them to contribute to type-2 inflammation independent of a humoral response [10]. The mediators released by mast cells are responsible for symptoms such as hives, itching, edema, and in the most extreme case, fatal systemic anaphylaxis. Thus, understanding the regulatory mechanisms that govern mast cell responses and homeostasis is critical given the potent consequences of mast cell activation. 

A number of cytokines have been identified that modulate mast cell function, including TGFβ. TGFβ signals through its receptors TGFβ receptor 1 (Tgfbr1) and 2, which dimerize and auto-phosphorylate in the presence of extracellular TGFβ. This leads to the phosphorylation of Smad2, which forms a complex with Smad3 and 4 that then translocates into the nucleus and acts as a transcription factor. Mast cells not only express Tgfbr1 and 2, but they also produce TGFβ as well as proteases that cleave inactive TGFβ into the active form. Growing evidence has demonstrated TGFβ to be a critical regulator of both mast cell development and function. IL-10 is another cytokine that has been demonstrated to play a role in mast cell activation. IL-10 signals through the IL-10 receptor (IL10r), which consists of a heterodimer between IL10r1 and IL10r2, leading to activation of the JAK/STAT signaling pathway as well the induction of suppressor of cytokine signaling (SOCS) proteins. Mast cells can release and respond to IL-10, and this cytokine has been reported to have both inhibitory as well as enhancing effects on IgE-mediated mast cell functions [11]. The alarmin IL-33, a member of the IL-1 family, was initially shown to enhance IgE-mediated mast cell functions. However, subsequent studies have found that long-term exposure to IL-33 can suppress mast cell activation. IL-37, another IL-1 family member, and IL-35, part of the IL-12 cytokine family, have also displayed mast cell suppressive abilities and may represent key regulatory pathways that control mast cell activation. This review will provide an overview of how these cytokine pathways regulate mast cell functions and the implications for allergic inflammation.

## 2. The Effects of Mast Cell-Derived TGFβ

Mast cells produce and respond to TGFβ; thus, there is the potential for both autocrine and paracrine regulation. The first description of mast cell production of TGFβ was in a study inspired by the positive correlation between mast cell numbers and TGFβ-induced fibrosis using dog mastocytoma cell lines [12]. In an in vitro study, IgE-induced mast cell mediators were shown to promote fibroblast proliferation, which could be inhibited with anti-TGFβ antibody treatment [13]. Furthermore, the same group showed that mast cell-derived TGFβ and TNFα induce cytokine production from fibroblasts [14]. In human cholestasis, increased mast cells were observed in the damaged liver tissue [15]. Using a bile duct ligation-induced liver injury mouse model, this group demonstrated that mast cell-deficient mice lacked liver fibrosis and damage [16]. Building on this finding, Kyritsi and colleagues showed that cholestasis related liver damage was dependent on mast cell-derived TGFβ [15]. Liver damage protection in mast cell-deficient mice was lost when they transferred TGFβ-producing mast cells but not TGFβ-deficient mast cells. 

In the context of allergic disease, mast cells have been found to promote regulatory T cell (Treg) function to induce oral immune tolerance to food antigens [17]. Using a murine model of oral immunotherapy (OIT), the accumulation of gastrointestinal Treg populations over the course of OIT was found to be dependent on mast cells. This induction required IL-2 production; however, a role for TGFβ was not evaluated. Evidence that mast cell-derived TGFβ also contributes to Treg upregulation was supported by another study where bone marrow-derived mast cells co-cultured with naïve T cells induced Treg differentiation, and this response could be inhibited by neutralizing TGFβ [18]. In addition, a correlative study presented that human germinal center FOXP3-expressing T cell numbers correlated positively with mast cell numbers [19]. However, a separate mechanism of mast cell–Treg interaction has also been shown through CD40 and OX40L in an in vitro model of OIT [20]. In addition, mast cell-derived IL-10 has been demonstrated to be critical for Treg-mediated peripheral tolerance in a murine autoimmune disease model [21]. Overall, these studies reveal that multiple mechanisms likely underly mast cell-mediated regulation of Treg development and function, including TGFβ production.

**Figure 1:**
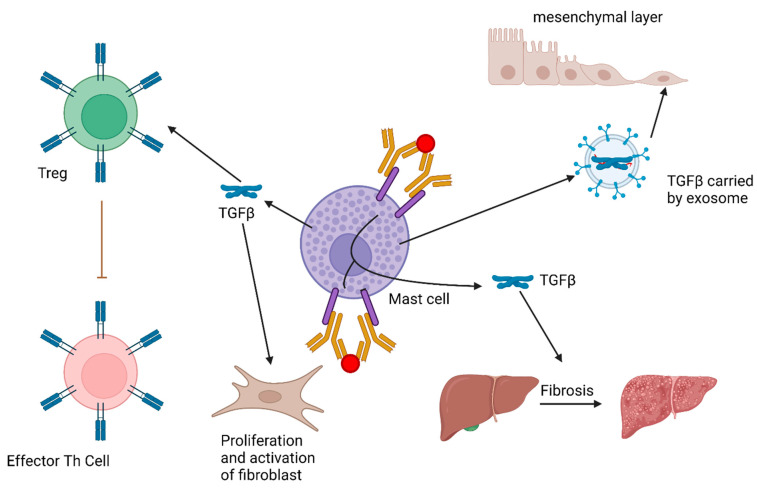
Currently Known Roles of Mast Cell (MC) Derived TGFβ. MC produce TGFβ upon IgE and antigen crosslinking. MC derived TGFβ has been shown to promote Treg function, which may be beneficial in controlling autoimmune and allergic inflammation. More detrimentally, it may also promote fibrosis, such as in the liver. Exosomes originating from MCs have been shown to carry active TGFβ that is able to induce Smad signaling in mesenchymal stem cells, suggesting MC derived TGFβ may have systemic effects.

Furthermore, human and mouse mast cell-derived exosomes were recently described to carry both active and inactive forms of TGFβ that induce endosomal mesenchymal stem cell Smad2/3 signaling [22]. The physiological implications of exosomal TGFβ are not fully understood; however, it is an intriguing area of study suggesting the possibility of systemic effects of mast cell-derived TGFβ. The role of mast cell-derived TGFβ is summarized in Figure 1.

## 3. The Role of TGFβ in Mast Cell Development and Survival

Several in vitro studies have highlighted the role of extracellular TGFβ in the development and survival of bone marrow-derived murine mast cells (BMMCs) and skin-derived human mast cells. Recombinant TGFβ treatment reduced expression of the receptor for the critical growth factor IL-3 in mature mouse and human mast cells, leading to mitochondrial damage and caspase-mediated apoptosis [23]. Furthermore, murine mast cells that differentiated in the presence of TGFβ showed reduced granule formation, degranulation, and cytokine production that occurred independent of Smad3 [24]. Importantly, viability was not affected in these experiments, in contrast to the apoptosis studies performed by the same research group, indicating important effects of both the dose of TGFβ applied and the differentiation stage of the mast cell culture. Interestingly, TGFβ treatment within the first 10 days significantly enhanced mast cell development, whereas suppression could be induced when adding recombinant TGFβ as late as day 10, suggesting a biphasic effect. 

TGFβ has been implicated to induce mucosal mast cells (MMCs) in vitro, despite showing an overall suppressive role in the studies discussed above. A study by Derakhshan and coworkers demonstrated that mast cell protease 1 (Mcpt1) producing mast cells that express integrinβ7, both markers of MMCs, are the primary mast cell subpopulation induced during Alternaria-mediated airway inflammation [25]. Furthermore, this population exhibited increased *Skil* gene expression, a known transcriptional target of TGFβ. BMMCs cultured with TGFβ showed similar upregulation of Mcpt1 and integrin B7 in comparison to BMMCs cultured in the absence of TGFβ. These results were replicated by another group that showed that Notch and TGFβ signaling cooperate to induce *Mcpt1* gene expression [26]. Furthermore, a separate group showed that TGFβ-mediated induction of Mcpt1 in BMMCs is facilitated by GATA and Smad transcription factors [27]. Taken together, these findings show TGFβ plays a multifaceted role in mast cell development and survival depending on developmental stage, TGFβ dose, and mast cell subset. 

## 4. The Role of TGFβ in Mast Cell Effector Function

Mast cell effector activities are mainly mediated by the release of potent preformed and newly synthesized mediators that lead to infection clearance or allergic symptoms; IgE-mediated mast cell functions are irrevocably tied to IgE-mediated allergic diseases. TGFβ was shown to inhibit the transcription and expression of FcεRI on mouse and human mast cells, thus potentially inhibiting IgE-mediated mast cell functions [28]. In addition, recombinant TGFβ suppressed the expression of c-Kit expression on human mast cells, and c-Kit signaling is known to enhance signaling downstream of FcεRI crosslinking [29]. Concurrently, the addition of recombinant TGFβ suppressed IgE-mediated cytokine production and degranulation from cultured human mast cells. However, in contrast to these suppressive roles, Lyons et al. demonstrated that recombinant TGFβ enhanced IgE-mediated IL-13 production [30]. Notably, these experiments were conducted in BMMCs cultured with IL-3 alone, whereas the previous studies differentiated BMMCs in the presence of both IL-3 and SCF. Furthermore, another study found variable effects of short-term TGFβ treatment on IgE-mediated responses and demonstrated that the genetic background of the mice influenced the response to TGFβ. Specifically, TGFβ suppressed IgE-mediated cytokine production in mast cells derived from C57BL/6 mice, whereas cytokine production was enhanced or unaffected by TGFβ treatment in 129/SV-derived mast cells [31]. In this study, TGFβ treatment was shown to reduce expression of Fyn and Stat5, which are involved in proximal FcεR1 signaling. Furthermore, the differences between C57BL/6 and 129/SV-derived mast cells were attributed to higher baseline levels of Fyn and Stat5B in 129/SV-derived mast cells. Additionally, a report examining the role of TGFβ in anaphylaxis in rats found that synthetic antisense TGF-β1 oligonucleotide treatment significantly repressed passive cutaneous anaphylaxis and histamine and TNFα release from mast cells [32], suggesting a positive role for endogenous TGFβ in IgE-mediated mast cell functions.

TGFβ has also been found to suppress IL-33 mediated mast cell functions [33]. TGFβ inhibited IL-33 mediated cytokine release from BMMCs that were derived from C57BL/6J, 129/SvJ, C3H/HeJ, and BALB/cJ mice. Mast cell- dependent IL-33 induced neutrophil recruitment was also abrogated following in vivo TGFβ treatment. Furthermore, TGFβ downregulated IL-33 and IgE mediated cytokine production from human skin-derived mast cells, and IL-33 mediated enhancement of IgE-induced cytokine production was also suppressed by TGFβ treatment. This response was associated with reduced activation of the proximal signaling molecules, Akt and Erk, and activity of the key transcription factors NFκB and AP-1 downstream of ST2 signaling. 

Collectively, the role of TGFβ in mast cell effector function remains to be fully elucidated since conflicting conclusions have been reported, most likely due to the complex nature of the TGFβ signaling pathway and its tight regulation. The role of TGFβ in mast cell function is summarized in Table 1.

## 5. Inferences on the Role of Mast Cell TGFβ Signaling in Allergic Diseases

The role of TGFβ in modulating mast cell functions in the context of allergic diseases is likely multifactorial and remains an area of active research. IL-9 producing MMCs have been implicated in driving food-induced anaphylactic reactions [34]. Interestingly, TGFβ was suggested to drive the development of IL-9 producing mast cells [34], although this phenomenon was only studied in vitro and it is not yet clear if this occurs in the small intestine microenvironment. In addition, small intestinal mast cells in mice lacking the TGFβ activator, integrin V6, displayed reduced expression of Mcpt1 and 2, both markers for MMCs, while tryptase expression was increased [35]. However, the clearance of the parasite *T. spiralis* in these mice was not affected. It is not known how IL-9 expression or food allergy-induced mast cell activation is altered in mice lacking integrin V6. However, a study examining the effects of loss of TGFβ specifically in Mcpt5 expressing mast cells found that total ovalbumin (OVA) specific IgE was increased, and OVA-induced anaphylaxis was augmented compared to controls [36]. Interestingly, since connective tissue mast cells express Mcpt5 while MMCs do not, these data suggest that connective tissue mast cell TGFβ signaling may have a humoral role in anaphylactic IgE production. As noted above, TGFβ has also been hypothesized to drive the maturation of MMCs in the airway during allergic inflammation [25]. In line with this and suggesting the possibility of an autocrine feedback loop, bronchial and transbronchial biopsies from patients with uncontrolled asthma displayed higher numbers of TGFβ+ mast cells compared to healthy volunteers and asthmatics whose disease was well-controlled [37].

In summary, the current literature suggests that TGFβ signaling in mast cells plays a pathogenic role in allergic conditions most likely by driving the differentiation of the mast cell subset, MMCs. However, mast cells may also play a favorable role in immune tolerance by producing TGFβ that drives Treg development and function. Importantly, the role of TGFβ in MMC differentiation has not been studied in vivo or in human systems, and further studies are needed to understand the implications of these current findings.

## 6. Updates on the Role of IL-10 in Mast Cell Regulation

The role of IL-10 signaling in mast cells has been reviewed recently [11]. IL-10 has pleiotropic effects on mast cell effector function, including the ability to promote IgE-mediated food allergy [38]. IL-10 deficient mice displayed decreased Th2 cytokine responses, loss of gastrointestinal mast cell accumulation, and reduced anaphylaxis in a food allergy model, which was reversed by the adoptive transfer of IL-10 expressing WT BMMCs but not IL-10 deficient BMMCs [38]. Furthermore, IL-10 signaling was shown to promote mir155 expression, which inhibited the phosphatase SOCS1, leading to exaggerated IgE-mediated signaling and functions [39]. Another recent study examined the effect of virus-derived IL-10 on mast cells. Para poxviruses such as red deer pox virus (RDPV) and Grey seal pox virus (GSPV) are known to encode an IL-10 like protein with high homology to human IL-10 that enables these organisms to evade immune-mediated clearance. Interestingly, RDVP and GSPV were found to induce murine mast cell proliferation as well as suppress monocyte pro-inflammatory cytokine production [40]. Thus, like TGFβ, IL-10 plays a multifactorial role in directing allergic and other immune responses by modulating mast cells. 

## 7. IL-35 Mediated Suppression of Mast Cells

IL-35 is in the IL-12 family and was first discovered in 2007 as a suppressive cytokine produced by T regulatory cells [41]. Since then, it has been recognized to potentially play a critical role in controlling allergic diseases. In a study conducted by Mohamed Shamji and colleagues, recombinant IL-35 suppressed IL-5 and IL-13 production from type-2 innate lymphoid cells (ILC2) isolated from pollen-allergic individuals following allergen stimulation [42]. Furthermore, IL-35 inhibited allergen-driven effector T cell proliferation ex vivo. This was further corroborated by Wehlong Lie and coworkers, who showed that ILC2s isolated from patients with allergic rhinitis exhibited decreased IL-35 receptor expression, and that recombinant IL-35 was able to suppress ILC2 effector functions [43]. Furthermore, IL-35 producing Tregs were decreased in the circulation of patients with allergic asthma, and allergen-driven Th2 cell induction was inhibited by IL-35 expressing Tregs in vitro. This inhibition could be reversed with the addition of anti-IL-35 [44]. Using an in vitro assay, this study also showed that naïve T cells from allergic individuals differentiated into IL-35 producing Tregs at a lower frequency compared to naïve T cells from healthy controls. IL-35 was also shown to inhibit Th17 responses in children with allergic rhinitis [45]. Further studies are warranted to understand the mechanism and consequences of IL-35 downregulation in allergic conditions. The role of IL-35 in mast cell effector function has been evaluated in a single study that showed IL-35 can suppress the release of histamine as well as mRNA expression of IL-6 and IL-17 from the human mast cell line HMC-1 following stimulation with phorbol myristate acetate (PMA) and the calcium ionophore, A23287 [46]. These effects were attributed to abrogation of signaling events since phosphorylation of p38, Jnk and Erk were repressed by IL-35, which are critical for mast cell functions. The mechanism of p38, Jnk and Erk suppression was not studied. Furthermore, it was found that patients with chronic spontaneous urticaria, a mast cell-driven disease, had decreased serum IL-35 levels that returned to normal after treatment [47]. Additional research is warranted to fully understand the role of IL-35 in regulating primary mast cells and allergic inflammation.

## 8. IL-37 Mediated Suppression of Mast Cells

IL-37 is a member of the IL-1 cytokine family. It binds to the IL18Rα receptor and IL-1 family decoy receptor, IL1R8, extracellularly. It also has intracellular functions, where it complexes with Smad3, which then translocates into the nucleus [48]. This anti-inflammatory cytokine has been demonstrated to suppress MyD88-mediated signaling downstream of ST2 and various toll like receptors (TLR) expressed on dendritic cells and T cell lineages. IL-37 has been primarily studied in the context of atopic skin conditions. A study by Weihua Li et al. demonstrated that intraperitoneal IL-37 injection ameliorated allergic contact dermatitis [49]. Since skin mast cells play a key role in this disease, they examined the role of mast cells as a potential mechanism and found that the protection was mediated by inhibition of mast cell IL-33 and IgE-induced function [49]. A second study found that IL-37 abrogated disease in a mouse model of psoriasis by inhibiting systemic and local cytokine levels that were driving a feedforward proinflammatory loop in this condition [50]. *In vitro*, IL-37 suppressed IL-6 and CXCL8 secretion from a human keratinocyte cell line (HaCaT), suggesting IL-37 can directly suppress pro-inflammatory cytokine production from cutaneous cells. Furthermore, it has been hypothesized that mast cell IL-37 signaling may be beneficial in CAR-T cell therapy by limiting the severity of the adverse reaction cytokine release syndrome, although this hypothesis requires further evaluation [51]. Considering that IL-37 was recently identified, additional roles for this cytokine in regulating mast cells and other cell types are likely to be discovered. Additionally, more studies are needed to understand the role of IL-37 in IgE and IL-33 mediated signaling and functions in mast cells.

## 9. Role of IL-33 in Mast Cell IgE-Mediated Functions

The receptor ST2 was discovered in 1989, and since then a growing body of research has revealed its importance in driving Th2 responses as well as innate cell responses [52,53,54]. In 2005, IL-33 was identified to be the ligand for ST2 [55]. Full-length IL-33 must be cleaved by caspases and other proteases to form mature, active IL-33 that can bind ST2 and trigger signaling through the MyD88 pathway. IL-33 was originally found to be an alarmin, released upon cellular damage and distress; however, several other mechanisms that contribute to IL-33 secretion have recently been recognized. 

ST2 is constitutively expressed on mouse and human mast cells at high levels. Recently, substantial effort has been devoted to elucidating the role of this signaling pathway in mast cell function. IL-33 was shown to induce mast cell release of the cytokines IL-13, IL-5, IL-6, IL-10, TNF, GM-SCF, CXCL8 and CCL1 [56]. IL-33 also stimulated secretion of eicosanoids PGD2 and LTC4, but did not trigger degranulation [56]. However, it was able to enhance IgE/antigen-mediated degranulation [57] as well as IgE/antigen-mediated cytokine and chemokine production [57,58]. In addition to promoting mast effector functions, IL-33 has also been demonstrated to prolong mast cell survival through the antiapoptotic molecule B-cell lymphoma-X large (BCLXL) [59].

Additional studies have provided insight into the role of IL-33 signaling in mast cells in allergic disease models. Leyva-Castillo and colleagues elegantly demonstrated that IL-33 released from damaged skin activated gut ILC2s to produce IL-4 and IL-13 that then drove expansion of gut mast cells, thereby promoting food-induced anaphylaxis [60]. Interestingly, in a model of OVA-induced lung inflammation, bronchoconstriction triggered by mast cell-derived serotonin was enhanced by IL-33, while allergen-specific IgE levels were unaffected [61]. The authors postulated that this effect was most likely the result of an IL-33 mediated increase in the storage and secretion of serotonin in mast cells, although this was not directly assessed. In a different model of OVA-induced lung inflammation, mast cell activation in the lungs was attributed to IL-33 mediated signaling, which was hypothesized to drive the Th17 response in this model. However, a direct effect of IL-33 on mast cell activation and the ability to drive Th17 differentiation was only evaluated in vitro [62].

More recently, several research groups have reported a negative regulatory role for IL-33 in regulating mast cell functions. In a papain-induced lung inflammation model, epithelium-derived IL-33 was shown to stimulate mast cell IL-2 production, which promoted Treg proliferation. The increase in Tregs led to reduced ILC2 effector function, blunting overall lung inflammation [63]. In addition, there is also evidence IL-33 can suppress FcεRI-mediated functions in a mast cell intrinsic manner. Several groups have shown that while short-term exposure to IL-33 enhanced IgE/antigen-mediated degranulation and cytokine production, long-term exposure led to suppression [64,65]. Mechanistically, reduced degranulation was tied to defective calcium mobilization, possibly secondary to inadequate phospholipase Cγ1 phosphorylation and Hck expression, which was MyD88-dependent [65]. Although this study did not find changes in FcεRI expression following chronic IL-33 exposure in CD34+ derived human mast cells and BMMCs, a separate study found that decreased IgE-mediated cytokine production was most likely due to decreased FcεRI expression in human lung-derived mast cells [64]. Furthermore, in an in vivo house dust mite (HDM) lung allergy model using mast cell-deficient mice that were reconstituted with ST2 knock out (KO) or ST2 WT BMMCs, it was demonstrated that attenuated lung hyperresponsiveness, as a result of decreased PGE2 production, could be attributed to IL-33/ST2 signaling in mast cells specifically [66].

**Figure 2:**
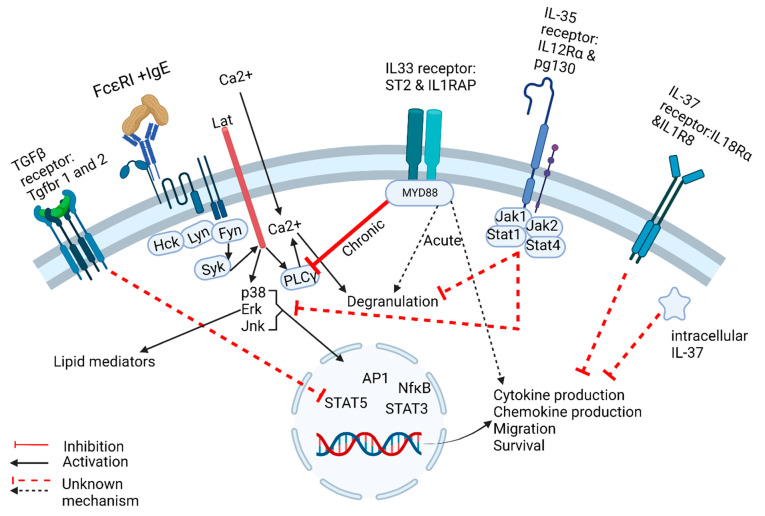
Currently Known Mechanisms of Cytokine-Mediated Regulation of MC Effector Functions. FcεRI signaling is initiated when IgE and specific antigen bind to its extracellular domain, recruiting Src family kinases Hck, Lyn, and Fyn leading to downstream activation of Syk, Lat, p38, Erk, and Jnk which leads to transcription factor activation and translocation into the nucleus to induce gene expression. Concurrently, Erk activation leads to the release of lipid mediators, and calcium influx downstream of Lat activation leads to degranulation. TGFβ signaling has been shown to suppress IgE mediated signaling most likely through Stat5 inhibition through an unknown mechanism. Acute IL-33 exposure enhances IgE mediated degranulation and cytokine production through an unclear signaling mechanism, most likely synergistically. Chronic IL-33 exposure inhibits IgE induced degranulation most likely by inhibition of PLCγ and calcium influx which is necessary for degranulation. IL-35 is thought to inhibit mast cell effector functions by suppressing p38, Erk and Jnk activation. IL-37 suppresses cytokine production by an unknown mechanism, which may involve signaling through its receptor and/or intracellular mechanisms.

Thus far, the preponderance of data suggest that acute IL-33 potentiates mast cell responses independently and augments antigen-specific responses. Interestingly, chronic exposure leads to hyporesponsive mast cells across multiple species. A better understanding of the cytokine-induced signaling pathways that lead to transcriptional changes and hyporesponsive mast cells without triggering degranulation requires further investigation and could provide attractive therapeutic targets. IL-33, IL-35, and IL-37 regulation of mast cells is summarized in Figure 2.

## 10. Conclusions

Mast cells have been shown to play a role in multiple biological processes such as protection against venom poisoning, parasitic, bacterial and viral infections, autoimmune conditions, wound healing, angiogenesis, fibrosis, and cancer, most likely through the production of cytokines and chemokines [67]. Mast cells are also a key effector cell in allergic reactions given their ability to release potent preformed mediators stored in granules upon IgE/FcεR1 activation. Although much is known about IgE-mediated signaling in mast cells, other regulatory pathways that govern mast cell effector functions are less understood. Furthermore, therapeutic options to inhibit mast cell activity are limited in their effectiveness, have hefty side effects, or are costly. A growing body of data underscore the role of cytokines as key regulators of mast cell function, providing an opportunity to harness these innate mechanisms as potential therapeutic targets. 

IL-10 signaling is well-studied but incompletely understood, and thus, further research may provide a clearer picture. IL-35 and IL-37 are both recently discovered regulatory cytokines and warrant further evaluation. Current studies of the role of these cytokines in controlling allergic inflammation and mast cell functions suggest they may be promising targets for inhibiting mast cell activation. The finding that the alarmin IL-33 may play a suppressive role in chronic allergic conditions is also interesting. This possibility requires further mechanistic studies in the context of allergic conditions and in humans in order to assess its therapeutic potential.

Although conflicting reports exist, overall there is clear evidence that TGFβ signaling has a broad and significant impact on mast cell effector functions in allergic diseases. This emerging area of research is deficient in comprehensive in vivo studies and human data. A clearer understanding of the role of TGFβ and other cytokines in mast cell function and allergic diseases may be beneficial to identify potential therapeutic targets for allergic disease prevention and treatment. 

MCs produce TGFβ upon IgE and antigen crosslinking. MC-derived TGFβ has been shown to promote Treg function, which may be beneficial in controlling autoimmune and allergic inflammation. More detrimentally, it may also promote fibrosis, such as in the liver. Exosomes originating from MCs have been shown to carry active TGFβ that is able to induce Smad signaling in mesenchymal stem cells, suggesting MC-derived TGFβ may have systemic effects.

FcεRI signaling is initiated when IgE and specific antigen bind to its extracellular domain, recruiting Src family kinases Hck, Lyn, and Fyn, leading to downstream activation of Syk, Lat, p38, Erk, and Jnk, which leads to transcription factor activation and translocation into the nucleus to induce gene expression. Concurrently, Erk activation leads to the release of lipid mediators, and calcium influx downstream of Lat activation leads to degranulation. TGFβ signaling has been shown to suppress IgE-mediated signaling, most likely through Stat5 inhibition through an unknown mechanism. Acute IL-33 exposure enhances IgE-mediated degranulation and cytokine production through an unclear signaling mechanism, most likely synergistically. Chronic IL-33 exposure inhibits IgE-induced degranulation, most likely by inhibition of PLCγ and calcium influx, which is necessary for degranulation. IL-35 is thought to inhibit mast cell effector functions by suppressing p38, Erk and Jnk activation. IL-37 suppresses cytokine production by an unknown mechanism, which may involve signaling through its receptor and/or intracellular mechanisms. 

## Figures and Tables

**Table 1:** Role of TGFβ in Mast Cell (MC) Effector Function.

Citation Number	Role of TGFβ	Conditions	Response	Species; Strain	MC Type
[28]	inhibitory	3 days of recombinant TGFβ1 prior to activation	FcεRI expression	murine; C57BL/6 and BL6 X 129	BMMCs grown with IL-3 and SCF
[29]	inhibitory	0, 1, 10, and 50 ng/mL TGF-β for 3, 5, and 7 days	c-kit expression; IgE mediated cytokine release and degranulation	human	skin derived cultured in SCF
[30]	augment	72 h of recombinant TGFβ1 including activation time	IgE mediated IL-13 secretion; SCF mediated IL-6 release	murine; C57BL/6	BMMC grown with IL-3
[31]	inhibitory	3 days of recombinant TGFβ1 prior to activation	IgE mediated cytokine secretion	murine; C57BL/6	BMMC grown in IL-3 and SCF
[31]	No effect to augment	3 days of recombinant TGFβ1 prior to activation	IgE mediated cytokine release	murine; 129/SV	BMMC grown in IL-3 and SCF
[31]	variable	3 days of recombinant TGFβ1 prior to activation	IgE mediated cytokine production	human	skin derived cultured in SCF
[32]	augment	in vivo, anti-TGFβ oligonucleotide treatment	IgE/antigen induced cutaneous anaphylaxis	rat	in vivo and in vitro rat peritoneal mast cells
[33]	inhibitory	3 days of recombinant TGFβ1 prior to activation	IL-33 mediated cytokine secretion and in vivo neutrophil recruitment	murine;C57BL/6J, 129/SvJ, C3H/HeJ, and BALB/cJ	BMMC grown in IL-3 and SCF
[33]	inhibitory	4 days of recombinant TGFβ1 prior to activation	IL-33 mediated cytokine production and IL-33 mediated enhancement of IgE induced cytokine production	human	skin derived cultured in SCF

## Data Availability

Not applicable.
